# Mechanobiology of hippocampal neurogenesis: directing neural stem cell fate through physical cues

**DOI:** 10.3389/fnmol.2026.1868741

**Published:** 2026-07-13

**Authors:** Irem Akyel, Emad Moeendarbary, Graham K. Sheridan

**Affiliations:** 1 School of Life Sciences, University of Nottingham, Nottingham, United Kingdom; 2 Department of Mechanical Engineering, University College London, London, United Kingdom

**Keywords:** dentate gyrus, extracellular matrix, hippocampus, neural stem cell, neurogenesis, Piezo1

## Abstract

Adult hippocampal neurogenesis (AHN), the generation of new neurons in the dentate gyrus of the hippocampus, is a dynamic and tightly regulated process essential for memory encoding, regulation of emotions, and cognitive flexibility. While the molecular and biochemical underpinnings of AHN have been studied extensively, recent advances have illuminated the pivotal role of mechanical forces in shaping neural stem cell (NSC) behavior. This perspective highlights the emerging field of hippocampal mechanobiology, examining how the physical properties of the neurogenic niche, such as extracellular matrix (ECM) stiffness and parenchymal viscoelasticity, act in concert with instructive biomechanical cues to govern NSC fate decisions. We explore the cellular machinery responsible for mechanosensing, including integrins, mechanosensitive ion channels, and cytoskeletal networks, and dissect the downstream signaling pathways, such as Rho GTPases and YAP/TAZ, that translate mechanical stimuli into transcriptional responses. We also review how physiological and pathological alterations in tissue mechanics influence neurogenesis and evaluate the therapeutic potential of biomaterials and pharmacological agents designed to modulate how cells interact with their mechanical microenvironment. By integrating mechanobiological principles into the study of AHN, we suggest new avenues for understanding brain plasticity and developing regenerative strategies for neurological disorders.

## 1 Neurogenesis and its microenvironmental regulators

The adult mammalian brain retains the capacity to generate new neurons, particularly within the subventricular zone (SVZ) of the lateral ventricles and the subgranular zone (SGZ) of the dentate gyrus (DG) (Altman and Das, 1965; Eriksson et al., 1998; Kuhn et al., 2018). These brain areas are specialized neurogenic niches that support lifelong neurogenesis (Dumitru et al., 2025). This discovery has fundamentally reshaped our understanding of brain plasticity (Aimone et al., 2014). Adult hippocampal neurogenesis (AHN) is now recognized as a tightly regulated, multi-stage physiological process. The key stages of AHN include the proliferation of neural stem cells (NSCs), their differentiation into neural progenitor cells (NPCs) and neuroblasts, and the subsequent maturation, migration, and synaptic integration of these newborn neurons into existing hippocampal circuits (Kempermann et al., 2004; Ming and Song, 2011; Overall et al., 2016). AHN is critical for hippocampal-dependent functions such as spatial learning, pattern separation, and mood regulation (Deng et al., 2010; Sahay et al., 2011). Conversely, its dysregulation has been implicated in a range of psychiatric and neurodegenerative disorders, including major depression, Alzheimer’s disease, and temporal lobe epilepsy (Jessberger et al., 2005; Kang et al., 2016; Toda et al., 2019). Historically, research aimed at understanding the multiple stages of AHN has focused on the identification of soluble biochemical cues, such as neurotransmitters, growth factors, and cytokines as well as downstream transcriptional regulators of protein synthesis (Li Y. et al., 2008; Chen L. et al., 2025). However, a growing body of evidence now supports the equally critical involvement of mechanical forces in shaping NSC behavior, including the biophysical properties of the neurogenic microenvironment (Conway and Schaffer, 2012; Sun et al., 2012). This emerging field of mechanobiology posits that cells are not simply passive recipients of biochemical signals. They are also active mechanosensors that continuously respond to and interpret physical cues such as extracellular matrix (ECM) stiffness, substrate topography, parenchymal viscoelasticity, fluid shear stress, and traction forces generated by neighboring cells (Engler et al., 2006; Janmey and Miller, 2011; Vining and Mooney, 2017; Comelles et al., 2023). Understanding how NSCs in the SGZ interpret these mechanical cues is essential for elucidating the full spectrum of regulatory mechanisms governing AHN, particularly in the context of aging, neurological disease, and brain injury (Babcock et al., 2021; Culig et al., 2022).

**FIGURE 1:**
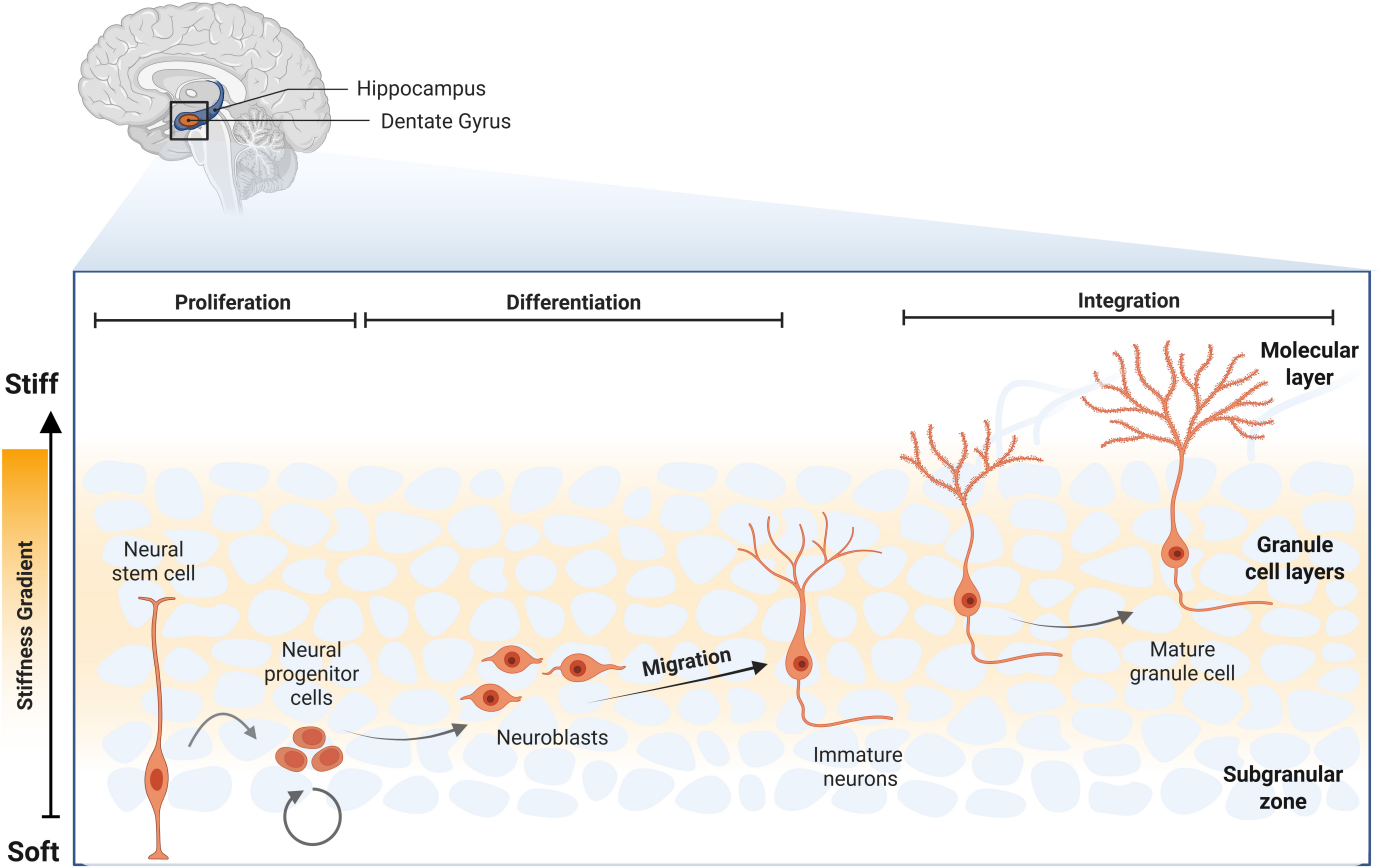
Schematic diagram briefly summarizing the stages of NSC development, maturation and integration into the hippocampal dentate granule cell network and how neurogenesis may, in part, be regulated by the stiffness gradient present within the SGZ neurogenic niche. Figure created using BioRender.com.

To build a deeper understanding of how mechanical cues modulate each phase of neurogenesis, advanced experimental techniques capable of probing the physical properties of the brain at both cellular and subcellular resolutions are required (Muhamed et al., 2017; Molnar and Manneville, 2025). Atomic force microscopy (AFM) has been instrumental in this pursuit. AFM facilitates high resolution contact-based mechanical mapping of *ex vivo* brain slices (Viji Babu and Radmacher, 2019). Results from micro-indentation experiments demonstrate that neighboring brain areas often display distinct mechanical landscapes at the cellular length-scale. This regional inhomogeneity in tissue stiffness is due to differences in ECM composition, myelination levels, and neural network wiring patterns. The complex physical microenvironment of the uniquely structured hippocampal formation is a prime example of this (Elkin et al., 2007, 2010, 2011; Antonovaite et al., 2018; Budday et al., 2020). Using AFM, we have shown that the dentate gyrus is softer than the CA1 region and that DG tissue stiffness increases with age. The dentate granule cell layer of 3-month old adult mice is approximately 180 Pa and increases to 311 Pa, on average, by 18-months of age (Hall et al., 2023). Therefore, the mechanical properties of the hippocampal dentate gyrus are not static; they evolve throughout brain development, postnatal maturation, aging, and are altered by injury and in disease states. Others have also shown that the neurogenic niche of the dentate gyrus is softer than adjacent brain structures (Morr et al., 2022). Specifically, the hilus and the outer granule cell layers that house more mature neurons exhibit slightly higher elastic modulus values that increase with age and brain maturation (Ryu et al., 2021). This creates a gradual stiffness gradient from the soft neurogenic SGZ to the stiffer outer layers of the DG. Therefore, mechanical gradients and topographical features could function as durotactic signals to newborn hippocampal neurons, guiding their integration into existing neural networks (Figure 1).

In addition to cytoarchitectural differences between brain regions, variations in electrical activity, blood flow and glial cell behavior can also interact to regulate the local mechanical landscape that NSCs experience (Mitchell et al., 2011; Antonovaite et al., 2020; Joy et al., 2023). For instance, neuronal activity and neurovascular coupling drive rapid, local changes in blood flow and vessel wall motion, thus producing short-lived shifts in interstitial pressure, fluid movement and tissue tension (Williams et al., 2023). Astrocytes, through water transport, calcium (Ca^2+^) signaling, and endfoot-mediated contact with vasculature, modulate blood vessel tone and the volume of perivascular spaces, thereby altering local fluid dynamics and the physical space around cells (Dunn et al., 2013; Zhang et al., 2019; Haidey et al., 2021; Walch and Fiacco, 2022; Sriram et al., 2024). Microglia can remodel the ECM and apply small traction forces as they change shape and survey the neurogenic niche (Bollmann et al., 2015; Nguyen et al., 2020; Wareham and Calkins, 2025). This may lead to subtle shifts in local tissue stiffness and trigger mechanotransduction signals in neighboring cells. Thus, actively proliferating NSCs and differentiating NPCs experience persistent activity-driven cellular forces that contain context-dependent and magnitude-specific information which guide the various stages of hippocampal neurogenesis (Moreira and Solá, 2024).

To date, one of the best characterized physiological modulators of AHN is physical exercise (van Praag et al., 1999; Yau et al., 2014; Inoue et al., 2015; Nokia et al., 2016; Gao et al., 2023). Not only does exercise upregulate brain-derived neurotrophic factor (BDNF); it also induces angiogenesis and ECM remodeling within the hippocampus (Sleiman et al., 2016; Chen et al., 2022; Maheu et al., 2025). Such structural changes potentially alter the mechanical properties of SGZ tissue creating a more permissive environment for NSC activation and neuronal differentiation. The inherent softness and dynamic compliance of the SGZ may thus be evolutionarily tuned to support the mechanical demands of neurogenesis in response to stimuli such as physical activity and environmental enrichment (Kempermann, 2016; Qiao et al., 2024; Huang et al., 2025). This article aims to provide a comprehensive perspective on the mechanobiology of hippocampal neurogenesis, synthesizing current knowledge on the mechanical properties of the neurogenic niche, the molecular machinery of mechanotransduction, and the implications of mechanical signaling for therapeutic modulation of neurogenesis.

## 2 The hippocampal neurogenic niche: a mechanically active microenvironment

The SGZ is composed of a diverse array of cell types, including radial glia-like NSCs (Type 1 cells), transit-amplifying progenitors (Type 2 cells), neuroblasts (Type 3 cells), astrocytes, microglia, oligodendrocytes, and vascular endothelial cells which are all embedded within a structurally dynamic extracellular matrix (Bond et al., 2015). It has been proposed that a compliant matrix supports the maintenance of quiescence, while subtle shifts in ECM stiffness or viscoelasticity activate Type 1 NSCs (Saha et al., 2008). Therefore, radial glia-like NSCs are thought to be maintained in a dormant state by a combination of biochemical inhibitors and mechanical constraints resulting from the inherent softness of the subgranular zone of the DG (Ryu et al., 2021). Upon activation, NSCs enter a proliferative phase, giving rise to transit-amplifying progenitors (Type 2 cells). Although the precise mechanical threshold that triggers the transition from quiescent to active has not yet been elucidated, *in vitro* studies employing tuneable hydrogels suggest that even modest shifts in substrate stiffness can bias NSC fate decisions. Soft substrates (0.1–1 kPa) have been shown to promote neuronal differentiation while stiffer environments (4–10 kPa) bias NSCs toward glial lineages (Saha et al., 2008; Leipzig and Shoichet, 2009). Importantly, experiments using physiologically relevant three-dimensional (3D) hydrogel-based culture systems corroborate these mechanosensitive features of NSCs (Chapla et al., 2024; Guo et al., 2026). Therefore, highly sensitive mechanically-gated ion channels that can detect functionally-relevant adjustments in tissue viscoelasticity are emerging as key regulators of hippocampal neurogenesis (Pathak et al., 2014; Mocciaro et al., 2025).

As NSCs exit the cell cycle and commit to a neuronal fate, they differentiate into neuroblasts and immature neurons (Type 3 cells). This transition is accompanied by dramatic changes in cell morphology, cytoskeletal architecture, and gene expression (Micheli et al., 2025). The cytoskeleton, particularly actin and microtubule networks, undergoes extensive remodeling during this stage and its mechanical coupling to the nucleus via the LINC (linker of nucleoskeleton and cytoskeleton) complex likely contributes to the transcriptional reprogramming required for neuronal differentiation (Momotyuk et al., 2025). Following differentiation, newborn neurons must migrate from the SGZ to the outer granule cell layers of the dentate gyrus and integrate into existing hippocampal circuits. This process is guided by both chemoattractant molecules, such as Reelin (Wang S. et al., 2018), and mechanical cues including cell-cell interactions and ECM stiffness gradients (Luque et al., 2016; Rodríguez-Iglesias et al., 2019; Nelson et al., 2020; Ryu et al., 2021). Cells cultured on substrates with stiffness gradients migrate toward zones of “optimal compliance,” demonstrating that migrating neurons are highly responsive to local tissue mechanics (Trichet et al., 2012; Wu et al., 2012; Kayal et al., 2020; Isomursu et al., 2022). Thus, *in vivo*, the mechanical inhomogeneity and topographical features of the hippocampal laminae may act as a roadmap to help migratory neuroblasts and immature neurons navigate their journey. Once positioned within the outer granule cell layers, successful network integration requires arborization of the dendritic tree which extends into the molecular layer of the DG. A polarized neurite also extends through the hilus to form the mossy fiber axon which connects to CA3 pyramidal cells (Rodríguez-Iglesias et al., 2019). These maturation processes occur over several weeks and during this time neuronal activity drives the formation of synaptic connections (Vivar and van Praag, 2013). Local mechanical forces generated by synaptic activity, for example the formation and decoupling of integrin adhesions or activity-induced interactions with neighboring glial cells, may provide positive feedback signals that reinforce circuit integration and functional neuron maturation (Kilinc, 2018; Minegishi et al., 2023). When this circuit integration process goes wrong it can lead to the formation of aberrant recurrent collaterals triggering the development of neurological disorders such as temporal lobe epilepsy (Goldberg and Coulter, 2013).

Taken together, recent findings underscore the importance of mechanical cues at every stage of adult hippocampal neurogenesis (Ryu et al., 2021; Guo et al., 2026). Leveraging the sensitivity of techniques such as AFM and traction force microscopy combined with tuneable hydrogels and live-cell imaging has been instrumental in uncovering the mechanobiological principles that govern NSC behavior (Shi et al., 2009; Spedden et al., 2012; Kjell et al., 2020; Kumarasinghe et al., 2022). As these techniques continue to evolve, they promise to yield deeper insights into how the physical properties of the neurogenic niche shape brain plasticity and may ultimately improve the development of therapeutic strategies and biomaterials aimed at enhancing neurogenesis in aging and disease.

## 3 Composition and mechanical properties of central nervous system extracellular matrix

In contrast to the ECM of peripheral tissues, which express high amounts of fibrous proteins like collagen type I and elastin, the ECM of the brain is a richly hydrated, non-fibrillar matrix. Collagen’s rigid structure provides tensile strength to tissues whereas elastin enables tissues to stretch and recoil back to their original shape (Trębacz and Barzycka, 2023; Hartley et al., 2025). The brain’s ECM, however, is composed primarily of (1) Hyaluronic acid (HA) which is a major glycosaminoglycan (GAG) that contributes to the hydration and viscoelasticity of the ECM, helping to trap water, reduce shear stress and provide cushioning to cells (Ruoslahti, 1996; Jensen et al., 2020; Weldy and Kumar, 2025). HA interacts with cell surface receptors, such as CD44 (cluster determinant 44) and receptor for hyaluronan-mediated motility (RHAMM), to regulate NSC proliferation and migration (Solis et al., 2012). (2) Chondroitin sulfate proteoglycans (CSPGs), including neurocan, brevican, and versican. CSPGs are abundant in perineuronal nets and modulate synaptic plasticity and neurogenesis (Bosiacki et al., 2019; Mencio et al., 2021). Their GAG chains are modified by sulfation, giving them a high negative charge density which attracts water and cations and contributes to the hydration and compressive stiffness of tissues (Han et al., 2011). Moreover, their sulfation patterns (e.g., 4-sulfated vs. 6-sulfated) can differentially regulate NSC fate (Foscarin et al., 2017; Fawcett and Kwok, 2022). (3) Tenascins, such as tenascin-C, are expressed during development and re-expressed in neurogenic niches and have been shown to modulate cell adhesion and migration (Chiquet-Ehrismann and Tucker, 2011; Chiquet-Ehrismann et al., 2014). Tenascin molecules can be stretched to several times their resting length due to the sequential unfolding of their repeating fibronectin type III (FnIII) domains. Their ability to rapidly refold means that they can act as mechanical shock absorbers to protect cells from excessive stress (Oberhauser et al., 1998; Imanaka-Yoshida and Aoki, 2014). (4) Laminins and fibronectin are glycoproteins that mediate cell-to-ECM adhesion via integrins and regulate cellular mechanotransduction, cytoskeletal re-organization, and cell polarity (Stukel and Willits, 2016; Cieśluk et al., 2022; Lim et al., 2022). Fibronectin fibrils are stretchable molecular springs that unfold in response to cellular traction forces which exposes binding sites for other ECM molecules (Klotzsch et al., 2009). Laminins spontaneously polymerize into organized meshworks that form structurally resilient mechanical linkages with the cellular cytoskeleton. By modulating the mechanical compliance of the cell-to-matrix interface, laminins directly mediate cellular mechanosensing of tissue rigidity and orchestrate downstream nuclear signaling (Barros et al., 2020). Therefore, the ECM of the subgranular zone of the dentate gyrus is not merely a passive scaffold but a dynamic and instructive component of the cellular microenvironment that functionally regulates NSC behavior (Riquelme et al., 2008; Devasthali et al., 2025). The activity-dependent remodeling of these matrix components via enzymatic breakdown (e.g., matrix metalloproteinases and hyaluronidases) or secretion of protein crosslinking enzymes (e.g., transglutaminase 2) ensures real-time adaptation of the neurogenic niche to everyday physiological demands such as learning and exercise (Urso et al., 2009; Liu et al., 2023), or to pathophysiological scenarios such as epilepsy and injury (Mizoguchi and Yamada, 2013; Shi et al., 2023). Thus, the biochemical composition, mechanical properties, and spatiotemporal plasticity of the brain’s ECM are fine-tuned to support adult hippocampal neurogenesis (Cope and Gould, 2019).

## 4 Time-dependent mechanical cues and vascular dynamics in the neurogenic niche

In addition to static stiffness, the brain’s ECM exhibits viscoelastic properties, meaning it displays elastic recoil and time-dependent relaxation under mechanical stress (Chaudhuri et al., 2020; Bergs et al., 2024; Liu Z. et al., 2024; Courbot and Elosegui-Artola, 2025). Recent work has demonstrated that faster stress relaxation rates, which closely resemble the reaction of native brain ECM, enhance neurite extension, reduce metabolic demand, and promote transcriptional programs associated with neuronal maturation (Roth et al., 2023; Li S. et al., 2025). These findings underscore the importance of temporal mechanical dynamics, not just absolute stiffness, in regulating NSC behavior. Brain tissue, including the dentate gyrus, behaves as a poro-viscoelastic material consisting of a soft, porous solid skeleton (cells and ECM) saturated by interstitial fluid (Su et al., 2023). Its low fibrillar collagen density, high water content, and enrichment in non-fibrillar components like hyaluronic acid and proteoglycans dictate the permeability and compliance of its solid scaffold, thereby regulating fluid redistribution and time-dependent load-sharing (Greiner et al., 2024; Liu Z. et al., 2024). Consequently, time-dependent responses such as stress relaxation and creep reflect a dual mechanism, i.e., fluid transport through the porous matrix (poroelastic relaxation) and the intrinsic viscoelasticity of the solid skeleton, both of which possess distinct physical origins and characteristic time-scaling. Within the subgranular zone (SGZ), these poro-viscoelastic properties may directly modulate neural stem cell behavior because NSCs are sensitive to both instantaneous matrix stiffness and the specific timescales of stress relaxation governed by matrix permeability and solid-phase viscoelasticity. Thus, changes in hydration or ECM composition, which may occur in age-related neurodegenerative disorders (Bonneh-Barkay and Wiley, 2009; Lepelletier et al., 2017; Vashisht et al., 2018), could alter time-dependent mechanical cues relevant to neurogenesis. A range of key physiological processes, including neuronal firing, astrocytic remodeling, and pulsatile blood flow, may also act as sources of continuous mechanical strain within the central nervous system (CNS) (Petzold and Murthy, 2011; Gomez-Cruz et al., 2024; Kasuba et al., 2024; Konig et al., 2025). Active neurons induce localized, transient tissue deformations and alter interstitial fluid dynamics, directly reshaping the micro-mechanical landscape of the SGZ (Tasaki and Byrne, 1990; El Hady and Machta, 2015; Ling et al., 2020; Zoraghi et al., 2021; Sætra et al., 2023; Jiang-Xie et al., 2024). Although these activity-dependent biomechanical shifts are subtle at the single-cell level, their cumulative spatiotemporal impact is sufficient to trigger mechanosensitive signaling pathways that direct neural stem cell behavior over long timescales (Rammensee et al., 2017).

**FIGURE 2:**
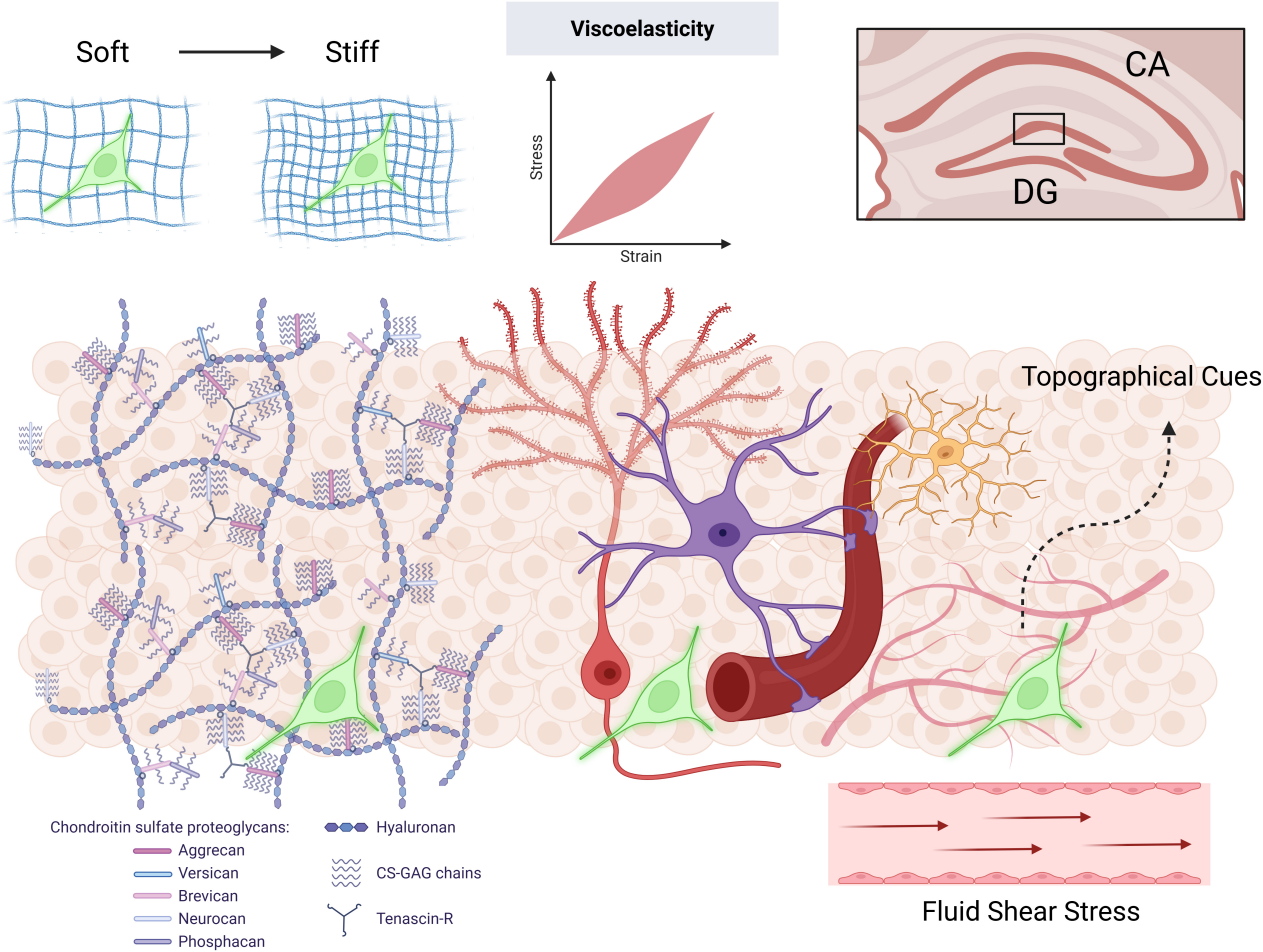
Schematic diagram describing some of the forces exerted on NSCs (green) within the hippocampal neurogenic niche. The viscoelastic ECM of the DG, along with topographical cues associated with cell body density, may act as instructive physical cues guiding migrating NSCs. Neuron-glial cell-to-cell contacts along with neuronal activity in the DG granule cell layers may also provide subtle mechanical cues to differentiating adult-born neurons. Finally, indirect forces such as fluid shear stress via capillary blood flow, or the subsequent release of angiocrine signaling molecules, may help to regulate hippocampal neurogenesis through modulation of the mechanical properties of the SGZ niche. Figure created using BioRender.com.

The vascular network within the SGZ introduces additional mechanical complexity to CNS tissue. Endothelial cells lining hippocampal capillaries experience fluid shear stress due to pulsatile blood flow, which is transmitted to adjacent pericytes, astrocytes, and NSCs (Palmer et al., 2000; Goldman and Chen, 2011; Dumont et al., 2017; Park et al., 2017). These cells express mechanosensitive ion channels, such as Piezo1 and TRPV4, which cluster with integrin adhesion complexes. This spatial organization enables them to respond to shear stress or matrix tension and activate intracellular signaling cascades that modulate gene expression, proliferation, and differentiation (Pathak et al., 2014; Maneshi et al., 2015; Schrimpf et al., 2017; Dessalles et al., 2021; Wan et al., 2023; Cibelli et al., 2024). Hemodynamic forces influence neurogenesis by altering the expression of angiocrine factors and by modifying the mechanical properties of the perivascular niche (Shen et al., 2004; Tan et al., 2016; Tata and Ruhrberg, 2018). These effects are particularly relevant in contexts such as exercise (Laughlin et al., 2008), which enhances cerebral blood flow and neurogenesis, and in hypertension, aging, or traumatic brain injury which disrupt vascular integrity and mechanical homeostasis (Iadecola and Davisson, 2008; Salehi et al., 2017; Santisteban and Iadecola, 2025). Taken together, such temporally patterned mechanical cues act in concert with the evolving viscoelastic properties of the ECM during development, aging, and disease to create a highly dynamic cellular microenvironment that orchestrates the progression of AHN (Figure 2). Understanding how NSCs integrate and translate these complex spatiotemporal mechanical signals remains a central challenge in the field and a promising avenue for therapeutic innovation (Dray et al., 2021).

## 5 Mechanosensing at the cellular level: how neural stem cells interpret mechanical cues

Neural stem cells within the hippocampal subgranular zone are equipped with a sophisticated array of mechanosensory systems that enable them to detect and respond to forces exerted by neighboring cells, blood vessels, or extracellular matrix remodeling (Stukel and Willits, 2016; Cavanaugh and Willits, 2025). This intricate system is fundamental to the regulation of adult hippocampal neurogenesis, influencing NSC proliferation, lineage specification, and synaptic integration (Keung et al., 2011; Mosher and Schaffer, 2018; Rezaei et al., 2026). NSCs also possess primary cilia which are solitary, immotile organelles that extend from the apical surface of most eukaryotic cells (Tong et al., 2014). Cilia are microtubule-based structures that function as cellular antennae by integrating mechanical, biochemical, and morphogenetic signals from the extracellular environment (Ringers et al., 2020). In NSCs, primary cilia are involved in regulating cell cycle progression, differentiation, and signal transduction through pathways such as Hedgehog, Wnt, and platelet-derived growth factor (PDGF) (Hasenpusch-Theil and Theil, 2021; Zhang et al., 2025). Although their role in hippocampal mechanosensing is not well defined, primary cilia are known to respond to fluid shear stress and substrate stiffness in other systems (Delaine-Smith et al., 2014; Spasic and Jacobs, 2017; Zhang et al., 2023). On that basis, they may help detect interstitial fluid flow, vascular pulsatility, or cell-generated forces within the SGZ, thereby influencing NSC behavior in a context-dependent manner. Mechanosensing machinery that have received more attention in recent years include integrin cell adhesion molecules, mechanosensitive ion channels, and the cytoskeletal network, all of which converge on intracellular signaling pathways that regulate stem cell proliferation, differentiation, and survival (Sun et al., 2012; Lv et al., 2015; Ferrai and Schulte, 2024). In the hippocampal neurogenic niche, mechanotransduction is not governed by a single linear cascade but rather by a tightly integrated framework involving calcium signaling, Rho GTPases, MAPK pathways, and the Hippo-YAP/TAZ axis (Yim and Sheetz, 2012; Rammensee et al., 2017; Kang et al., 2020; Baek et al., 2022). Together, these molecular systems enable NSCs to adapt to the mechanical properties of their environment and maintain the balance between self-renewal and differentiation.

### 5.1 Integrins: transducing ECM mechanics into intracellular signals

Integrins are heterodimeric transmembrane receptors composed of α and β subunits that serve as critical mediators of cell-ECM adhesion and mechanotransduction (Sun et al., 2016). On the extracellular side, integrins bind to ECM ligands such as fibronectin, laminin, and collagen, while their cytoplasmic domains connect to the actin cytoskeleton via adaptor proteins including talin, vinculin, and focal adhesion kinase (FAK) (Brakebusch and Fässler, 2003; Bachmann et al., 2019). Hippocampal NSCs and their progeny express multiple integrin isoforms (e.g., α1β1, α5β1, α6β1), which are dynamically regulated in response to ECM composition and stiffness (Brooker et al., 2016; Jaudon and Cingolani, 2024). Mechanical cues, such as increased substrate stiffness, promote integrin clustering and focal adhesion formation leading to activation of FAK and Src family kinases. These, in turn, regulate downstream pathways involved in cell cycle progression, survival, and lineage commitment (Choquet et al., 1997; Paszek et al., 2009; Provenzano and Keely, 2011). Conversely, softer substrates reduce integrin-mediated tension, favoring stem cell self-renewal and maintenance of the pluripotent niche (Chowdhury et al., 2010; Gerardo et al., 2019). In addition to ECM sensing, integrins also mediate cell-cell mechanical interactions, particularly between NSCs and niche-resident astrocytes, endothelial cells, and microglia (Mueller et al., 2006; Hayakawa et al., 2014; Morimoto et al., 2024). These interactions contribute to the mechanical integrity of the SGZ and modulate NSC behavior through juxtacrine signaling and mechanical feedback loops. Ultimately, by integrating these diverse physical cues, integrins act as the primary mechanical rheostat, translating the complex architecture of the neurogenic niche into the biochemical signals that dictate NSC fate (Chen et al., 2013; Lim et al., 2022).

### 5.2 Mechanosensitive ion channels: direct transducers of force

Mechanosensitive ion channels open in response to mechanical stimuli, providing a rapid, direct mechanism for cells to transduce external or internally-generated forces into electrical and biochemical signals. Piezo channels, particularly Piezo1, have emerged as key players in hippocampal mechanotransduction and are expressed on NSCs, astrocytes, neurons and endothelial cells (Velasco-Estevez et al., 2020a; Velasco-Estevez et al., 2020b; Nourse et al., 2022; Zong et al., 2023; Chen I. T. et al., 2025; Jia et al., 2025; Nourse and Pathak, 2017). Piezo1 is a large non-selective rapidly-adapting cation channel that is activated by membrane tension and facilitates the influx of calcium (Ca^2+^) and other cations (Liu et al., 2025). Ca^2+^ serves as a universal second messenger in mechanotransduction, linking mechanical stimuli to a broad range of intracellular responses (Jones and Nauli, 2012). Falleroni et al. (2022) showed that even pico-Newton-scale forces can elicit localized Ca^2+^ transients in hippocampal neurons, suggesting that the neurogenic niche may be exquisitely sensitive to mechanical fluctuations. In hippocampal NSCs, Piezo1 activation has been shown to regulate proliferation, differentiation, and survival. For example, Mocciaro et al. (2025) demonstrated that mechanical stretch injury activates Piezo1 in NSCs, and its inhibition via GsMTx4 peptide or siRNA promotes neurogenic differentiation. Similarly, Chi et al. (2022) found that astrocytic Piezo1 channels regulate calcium signaling and ATP release and that conditional deletion of Piezo1 in astrocytes impairs long-term potentiation (LTP), adult neurogenesis, and learning and memory behaviors in mice. These results highlight the key role of Piezo1 in mechanical signal integration and circuit-level synaptic plasticity (Csemer et al., 2024). Ca^2+^ influx via mechanosensitive channels activates downstream effectors such as CaMKII, protein kinase C (PKC), and calcineurin, which regulate transcription factors and cytoskeletal remodeling (Johnson et al., 2024; Lan et al., 2024; He et al., 2025; Li Z. et al., 2025; Liu et al., 2025; Tan et al., 2025). These Ca^2+^ signals also intersect with mitogen-activated protein kinase (MAPK) pathways and YAP/TAZ transcriptional coactivators, contributing to a multimodal mechanotransduction network (Bakhshandeh et al., 2023). Other mechanosensitive channels, such as TRPV4, may also contribute to NSC mechanosensing, although their specific roles in hippocampal neurogenesis remain less well defined.

### 5.3 Rho GTPases and MAPK signaling cascades: translating force into NSC fate decisions

The Rho family of small GTPases, including RhoA, Rac1, and Cdc42, are master regulators of the actin cytoskeleton and play a central role in cellular mechanotransduction (Nobes and Hall, 1995; Hoon et al., 2016; Burridge et al., 2019). These GTPases act as molecular switches that respond to mechanical stimuli by modulating actin filament organization, stress fiber formation, and focal adhesion dynamics. Increased ECM stiffness or mechanical stretch activates RhoA, which promotes actomyosin contractility via its downstream effector ROCK (Rho-associated kinase) (Zhang et al., 2018; Tu et al., 2022). This generates intracellular tension that feeds back into mechanosensitive pathways such as the Yes-associated protein (YAP) and transcriptional coactivator with PDZ-binding motif (TAZ) axis, robustly driving proliferation and mechanical fate selection. Conversely, inhibition of RhoA or ROCK reduces cytoskeletal tension, facilitating NSC differentiation (Gu et al., 2013). The spatiotemporal regulation of Rho GTPase activity is essential during neurogenesis. Specifically, RhoA activity is required for the maintenance of apical-basal polarity and the orientation of the cleavage plane during asymmetric division of NSCs (Herzog et al., 2011), ensuring the generation of neurogenic progeny. While RhoA governs the initial division, Cdc42 becomes paramount in the post-mitotic stage, where it drives neurite initiation and coordinates the directional migration of young neurons toward their functional targets (Garvalov et al., 2007). These processes are tightly coupled to mechanical inputs, highlighting the role of Rho GTPases as mechanical integrators that link extracellular forces to intracellular architecture and fate decisions.

Downstream of these mechanical integrators are the MAPK pathways, including ERK1/2, JNK, and p38, which act as biochemical transducers that respond to mechanical stress via integrin engagement and cytoskeletal tension (Aikawa et al., 2002; Hoffman et al., 2017; Crozet and Levayer, 2023). These signaling pathways regulate NSC proliferation, differentiation, and stress adaptation, often acting in synergy with Ca^2+^ fluctuations and YAP/TAZ-activated pathways (Di et al., 2023). Mechanical triggering of MAPKs occurs through focal adhesion complexes, where integrin clustering and force-dependent kinase activation initiate transcriptional programs. For example, ERK signaling is activated by substrate stiffness and modulates NSC proliferation (Wang et al., 2009; Farahani et al., 2021). The cross-talk between calcium signaling, Rho GTPases, and MAPK pathways ensures a robust and context-specific integration of mechanical cues, enabling NSCs to adapt to the dynamic mechanical landscape of the hippocampal niche (Hoon et al., 2016; Li L. et al., 2025).

### 5.4 Cytoskeletal coupling to the Hippo-YAP/TAZ pathway

**FIGURE 3:**
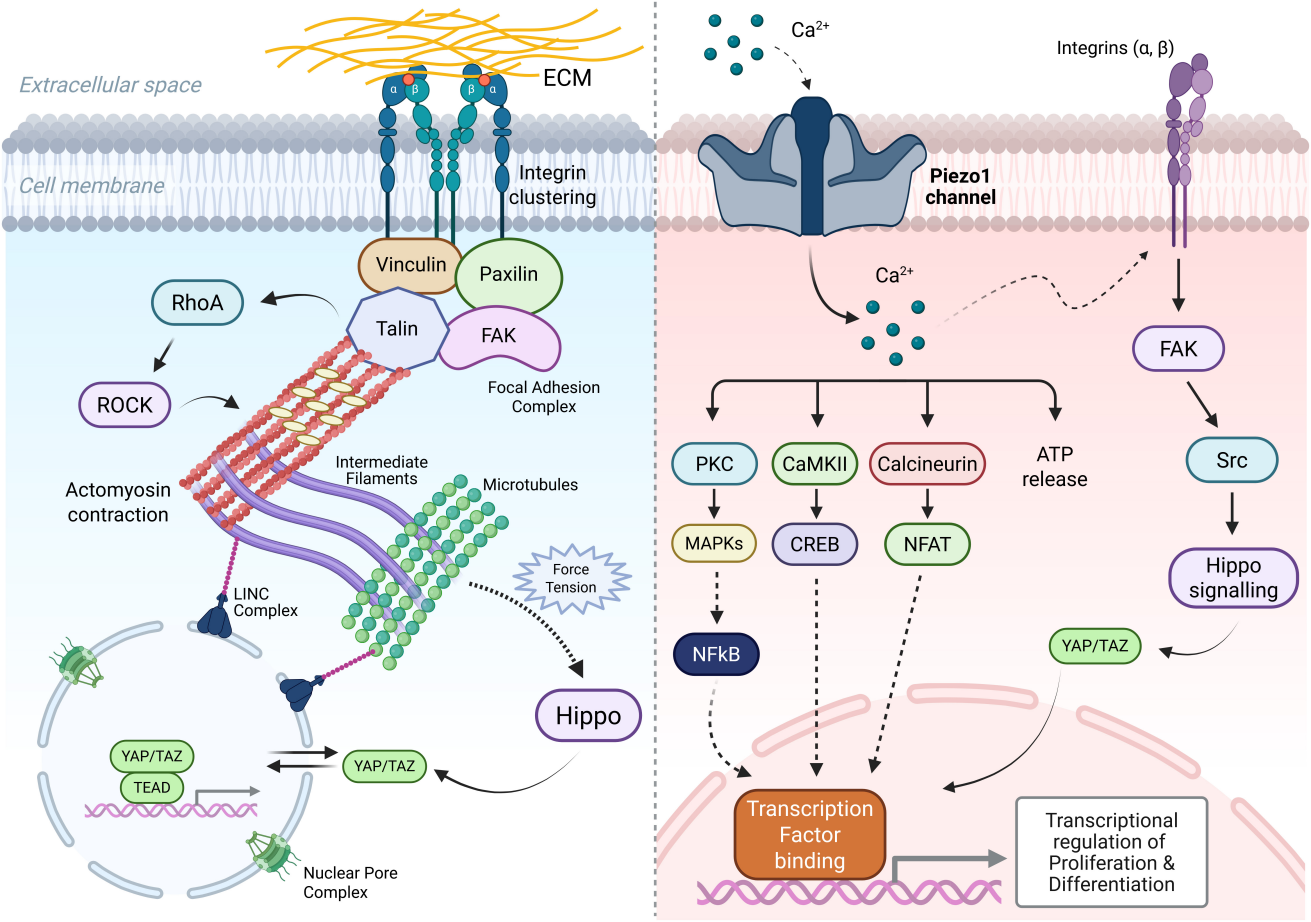
Schematic diagram summarizing some key mechanotransduction-mediated signaling pathways in neural stem cells. On the left hand side, ECM elasticity is sensed by the NSC cytoskeleton via integrin cell adhesion molecules and focal adhesion complexes. Actomyosin contractility can regulate the Hippo signaling pathway components and modulate transcription via YAP/TAZ translocation to the nucleus. On the right hand side, membrane deformations or fluid shear stress can activate the opening of mechanosensitive ion channels, such as Piezo1, on NSCs. The subsequent Ca^2+^ influx can lead to activation of integrin-mediated signaling or kinase activity resulting in transcriptional regulation of proliferation or differentiation. Figure created using BioRender.com.

Integration of ECM components, focal adhesion complexes, mechanosensitive ion channels, and the cytoskeletal network constitutes a highly coordinated mechanotransduction system (Figure 3). This network enables NSCs to decode the physical properties of their microenvironment and to precisely control intracellular signaling in response to shifts in tissue mechanics (Kalukula et al., 2025). The cytoskeleton, comprising actin filaments, intermediate filaments, and microtubules acts as both a structural scaffold and a functional conduit for mechanical signal transmission (Fletcher and Mullins, 2010; Hohmann and Dehghani, 2019). Specifically, actin stress fibers link focal adhesions to the nuclear envelope via the LINC complex, enabling force transmission from the ECM to the nucleus (Lityagina and Dobreva, 2021). This mechanical coupling influences nuclear shape, chromatin organization, and gene expression (Goelzer et al., 2021). Mechanical signals transmitted through the cytoskeleton can also modulate the Hippo signaling pathway. Recent evidence suggests that downstream effectors of the Hippo pathway, YAP/TAZ, play pivotal roles in NSC mechanotransduction (Han et al., 2015). For instance, when cells are exposed to conditions of elevated mechanical tension, such as increased ECM stiffness or RhoA-mediated F-actin polymerization and ROCK-dependent myosin II contractility, YAP/TAZ translocate to the nucleus. There, they bind to TEAD transcription factors to induce transcriptional programs that regulate stemness, cell cycle progression, and anti-apoptotic signaling (Zhao et al., 2008; Dupont et al., 2011; Tamm et al., 2011). Conversely, in soft or compliant environments, mechanical signaling shifts to alternative pathways. Low-stiffness matrices activate RAP2 GTPase and the ARID1A–SWI/SNF chromatin-remodeling complex upstream of YAP/TAZ, thus integrating physical cues at both the cytoplasmic and epigenetic levels (Chang et al., 2018; Meng et al., 2018). Concurrently, the core Hippo kinases MST1/2 and LATS1/2 phosphorylate YAP/TAZ, triggering their cytoplasmic retention or proteasomal degradation, thereby limiting unconstrained proliferation and promoting neuronal differentiation (Zhang et al., 2012; Panciera et al., 2016; Dobrokhotov et al., 2018; Pardo et al., 2025). As nuclear mechanosensors, YAP and TAZ integrate signals from ECM stiffness, cell shape, and cytoskeletal tension to regulate both transient and long-term transcriptional profiles (Dupont, 2016; Panciera et al., 2017). This mechanosensitive axis is critical for maintaining the structural and functional plasticity of the neurogenic niche (Blasco-Chamarro et al., 2025).

### 5.5 Stem cell mechanosensory divergence and fate determination

Interestingly, the cellular response to matrix rigidity is highly tissue-specific. *In vitro* studies utilizing two-dimensional substrates demonstrate that matrix compliance can instruct neural stem cell fate, although specific lineage trajectories depend heavily on hydrogel chemistry and the biochemical composition of culture media. On ultra-soft 2D hydrogels mimicking the mechanical properties of native brain tissue (0.1–1 kPa), NSCs preferentially exit the cell cycle and commit to a neuronal lineage. Conversely, intermediate stiffness profiles (1–4 kPa) provide the optimal mechanical feedback to sustain stemness, with NSC proliferation peaking around 3.5 kPa (Saha et al., 2008; Leipzig and Shoichet, 2009). Elevating substrate stiffness beyond this boundary (4–10 kPa) directs NSC differentiation toward a predominantly glial cell fate. This mechanical sensitivity contrasts with mesenchymal stem cells (MSCs) which enter into a state of quiescent cell-cycle arrest on ultra-soft matrices (0.25 kPa) (Winer et al., 2009). Instead, MSCs require progressively stiffer kilopascal substrata to execute distinct structural differentiation programs. For instance, myogenesis occurs on intermediate compliance (8–17 kPa) and osteogenesis requires rigid substrates (25–40 kPa) (Engler et al., 2006). More recent studies have developed 3D biomimetic hydrogels that introduce omnidirectional spatial constraints and matrix permeability parameters. These tuneable 3D microenvironments are useful for simulating postnatal maturation of the hippocampus where gradual stiffening of the dentate gyrus and hilar regions coincides with a marked decline in NSC numbers and neurogenic activity in the SGZ (Ryu et al., 2021). This suggests that the mechanical maturation of hippocampal tissue may act as a developmental brake on neurogenesis, potentially contributing to the natural decline in cognitive flexibility and synaptic plasticity as we age (Amelchenko et al., 2023; Navakkode and Kennedy, 2024; Hanushek et al., 2025). Mechanistically, this progressive increase in ECM rigidity (112, 345, and 1048 Pa) activates mechanosensitive ion channels, such as Piezo1, on resident NSCs. Persistent Piezo1 signaling induces a mechanical stress response that triggers chronic intracellular Ca^2+^ influx and downstream nuclear translocation of YAP/TAZ. This mechanosensitive cascade drives transcriptomic events that upregulate p16 and p21, cyclin-dependent kinase inhibitors that induce cellular senescence and cause depletion of the active stem cell reservoir. However, encapsulating aged NSCs within the soft 3D hydrogels (112 Pa) that mimic the young postnatal SGZ effectively silences this aging phenotype. By restoring a compliant physical microenvironment, these 3D matrices deactivate Piezo1, lower intracellular tension, and downregulate senescent checkpoints, thereby rescuing the neurogenic potential of aged NSCs and restoring youthful self-renewal (Guo et al., 2026).

## 6 Harnessing mechanobiology to promote neurogenesis and functional regeneration in neurological disorders

The emerging understanding of mechanobiology as a central regulator of adult hippocampal neurogenesis may have important implications for the development of novel therapeutics. Many neurological disorders are characterized by profound maladaptive changes in tissue mechanics ranging from cavitation and tissue softening (Ghuman et al., 2018; Baumann et al., 2020; Hall et al., 2023) to fibrosis, plaque formation and increased local stiffness (Wang L. et al., 2018; Urbanski et al., 2019; Hu et al., 2023). In neuroinflammatory disorders such as epilepsy and stroke, the ECM undergoes significant remodeling, including increased deposition of chondroitin sulfate proteoglycans and altered expression of matrix metalloproteinases (MMPs) (Kim and Han, 2006; Bronisz and Kurkowska-Jastrzębska, 2016; Patel et al., 2024). These proteolytic changes can disrupt the viscoelastic properties of the subgranular zone of the DG and impair NSC proliferation and differentiation, thus limiting endogenous repair mechanisms and accelerating cognitive deficits (Delgorio et al., 2021; Rai et al., 2022; Tarbox et al., 2025). Strategies that manipulate the mechanical properties of the neurogenic niche or target the intracellular pathways that transduce mechanical signals into cell fate decisions may aid in restoring or enhancing neurogenesis in the aged, injured, or diseased brain (Rocha et al., 2022; Velikic et al., 2024).

### 6.1 Biomaterials as regenerative scaffolds for ischemic stroke

A defining hallmark of ischemic stroke is the initial phase of cytotoxic oedema, cell death, and loss of brain tissue structure and integrity (Han et al., 2023). Over time, this is followed by cavitation, glial scarring, and tissue softening leading to inhomogeneous mechanical landscapes within the infarct zone and the surrounding penumbra (Michalski et al., 2015). Combined with hypoxia and inflammation, these mechanical disturbances severely impair neurogenesis, both locally and in connected brain regions such as the hippocampus (Osborne et al., 2024). The altered cerebrovascular dynamics that accompany stroke together with mechanical disruption of the neurogenic niche will likely interfere with NSC migration and integration, further limiting functional recovery. Therefore, regenerative therapies that aim to restore the mechanical properties of ischemic brain tissue, in addition to stimulating a pro-neurogenic biochemical milieu of growth factors, will likely have a synergistic effect in promoting functional regeneration after a cerebrovascular stroke (Collins et al., 2022; Geng et al., 2026; Rahman et al., 2021). A promising translational avenue involves the use of biomaterials, particularly hydrogels, to engineer synthetic microenvironments that recapitulate the mechanical and biochemical properties of the native SGZ of the DG (Wang M. et al., 2025). Hyaluronic acid (HA), polyethylene glycol (PEG), or gelatin methacryloyl (GelMA)-based hydrogels can be precisely tuned in terms of stiffness and viscoelasticity to match the mechanical profile of the healthy hippocampal niche (Kim and Choi, 2019). Studies have shown that NSCs cultured on soft, viscoelastic substrates exhibit enhanced proliferation and neuronal differentiation, with improved neurite outgrowth and synaptic maturation (Liang et al., 2021). For example, Roth et al. (2023) demonstrated that HA and elastin-like protein-based hydrogels with low stiffness (approx. 800 Pa) and fast stress relaxation rates promoted neurogenic differentiation of encapsulated human neural progenitor cells. These findings are consistent with the broader literature showing that mechanical compliance and dynamic remodeling of the ECM are critical for supporting neurogenesis (Ma et al., 2008; Sart et al., 2016; Vieira et al., 2018).

The development of injectable hydrogels further enhances the clinical relevance of these bio-inspired materials (Guo et al., 2025; Parvin et al., 2025). Moreover, topographical features embedded within soft biomaterials, such as patterned ridges, microgrooves, or aligned nanofibres can also serve to guide NSC migration, orientation, and axonal pathfinding (Yao et al., 2016; Liu et al., 2021). Nguyen et al. (2017) showed that aligned electrospun nanofibres can direct neurite extension and enhance synaptic connectivity in rat spinal cord injury, suggesting that topographical cues may be leveraged to improve the integration of newborn neurons into existing hippocampal circuits. Interestingly, 3D hydrogels encapsulating magnetically-aligned or electrically-conductive nanofibres may enhance neuronal maturation and axonal pathfinding *in vivo* (Johnson et al., 2019; Yao et al., 2024). Thus, the minimally invasive delivery of hydrogels to an ischemic infarct zone or site of neurodegeneration could provide a mechanically supportive scaffold that conforms to irregularly-shaped cavities and evolves over time as tissue repair and neurogenesis progresses (Nih et al., 2016; Jiang et al., 2023; Li H. et al., 2025). Moreover, injectable hydrogels could be used to deliver cells or biologic therapies to the damaged area to enhance innate functional regeneration processes of CNS tissue (Wang et al., 2012; Wang et al., 2013; Tuladhar et al., 2020).

### 6.2 Therapeutic avenues for traumatic CNS injury

In traumatic brain injury (TBI) or spinal cord injury (SCI), the initial mechanical insult is characterized by rapid stretch, compression, contusion and shear forces which can directly damage NSCs and their microenvironment (Rola et al., 2006; Zhou et al., 2012; Falnikar et al., 2018). This is followed by a secondary phase of injury involving blood-brain barrier (BBB) breakdown, inflammation, and glial scar formation (Simon et al., 2017; Amlerova et al., 2024; Bhatt et al., 2024). We have previously shown that, up to 3 weeks post-SCI or cortical stab wound injury in rats, the glial scar that forms is softer than surrounding CNS tissue (Moeendarbary et al., 2017). Glial scars are rich in inhibitory molecules, such as CSPGs, which further suppress neurogenesis (Ohtake and Li, 2015; Galindo et al., 2018; Bradbury and Burnside, 2019). Chronic post-injury changes, including persistent ECM remodeling and astrocytic hypertrophy, have been argued to create a mechanically hostile environment that limits the potential for endogenous repair (Tran et al., 2018; Cooper et al., 2020). On the other hand, there is evidence to suggest that the glial scar creates a mechanical and biochemical environment that enhances neuronal repair and that techniques aimed at ablating the glial scar inhibit functional recovery (Anderson et al., 2016). Therefore, regenerative strategies for TBI and SCI could utilize soft, functionalized hydrogels to deliver NSCs directly to the glial scar. Designing these matrices to gradually release ECM-modulating enzymes creates local stiffness gradients, providing physical cues that could trigger the differentiation of stem cells into mature neurons.

In parallel, pharmacologically targeting mechanotransduction pathways offers a complementary approach to modulating neural stem cell behavior. Mechanosensitive ion channels, such as Piezo1, have emerged as key regulators of NSC fate and are activated by the mechanical stress of TBI and intracerebral hemorrhage (ICH). Inhibition of Piezo1 post-TBI promotes the differentiation of hippocampal NSCs to mature neurons rather than glial cells (Mocciaro et al., 2025). Moreover, blocking neuronal Piezo1 channels post-ICH reduces brain oedema and enhances functional recovery and memory in mice (Qi et al., 2024), possibly by reducing cytotoxic levels of calcium influx and maladaptive downstream inflammatory signaling. However, in otherwise healthy mice, deletion of astrocytic Piezo1 reduces hippocampal volume and brain weight and impairs adult neurogenesis, whilst overexpression of Piezo1 in astrocytes enhances LTP and memory performance (Chi et al., 2022). These cell type-specific results emphasize the delicate balance that mechanosensitive channel activity exerts in the regulation of hippocampal NSC proliferation and differentiation. Similarly, pharmacological regulation of the Hippo-YAP/TAZ pathway, which integrates mechanical cues from the ECM and cytoskeleton, represents a potential target for enhancing neurogenesis. Compounds that promote nuclear localization of YAP/TAZ may enhance NSC proliferation and self-renewal, while those that favor cytoplasmic retention could facilitate neuronal differentiation (Rammensee et al., 2017; Han et al., 2020; Lavado et al., 2021; Seo et al., 2022; Luo et al., 2025). However, given the pleiotropic roles of YAP/TAZ in other tissues, including their involvement in oncogenesis (Kim and Nam, 2025), therapeutic strategies must be precise, context-specific and cell type-selective. Alternative pharmacological tools could include the use of cytoskeletal modulators, such as Rho-associated kinase (ROCK) inhibitors, which can alter intracellular tension and focal adhesion dynamics (Amano et al., 2010; Christie et al., 2013), thereby influencing mechanosensitive signaling cascades. Used in combination with neurotherapeutics that regulate ECM remodeling, such as matrix metalloproteinase inhibitors or enhancers (Wójcik-Stanaszek et al., 2011), BBB-permeant pharmacological agents may prove to be useful adjuncts for softening fibrotic tissue and restoring a more permissive mechanical environment for neurogenesis to thrive.

### 6.3 Correcting aberrant neurogenesis in temporal lobe epilepsy

Temporal lobe epilepsy (TLE) is increasingly recognized as a disorder in which pathological neuronal activity co-evolves with long-lasting changes in hippocampal tissue architecture and cell mechanics. Recurrent seizures can cause sustained cytoskeletal remodeling, reactive gliosis, and vascular dysfunction, which collectively indirectly alter the mechanical microenvironment experienced by neurons and NSCs (Scharfman, 2019; Liu X. et al., 2024; Dalir et al., 2025, *preprint*; Onat et al., 2025). These cellular changes occur alongside profound ECM remodeling, including altered expression of tenascins, CSPGs, and integrin adhesion complexes. Consequently, the hippocampal microenvironment progressively diverges from its physiological biomechanical state, which normally restricts aberrant plasticity to maintain circuit stability (Dityatev, 2010; Pitkänen et al., 2014). These pathophysiological hallmarks of TLE, namely dysregulated ECM and SGZ niche viscoelasticity, fundamentally disrupt adult hippocampal neurogenesis and bias NSC fate decisions, migration trajectories, axonal targeting, and synaptic stabilization. The resulting improper integration of newborn neurons into the hippocampal network, combined with seizure-induced loss of local interneurons, drives aberrant mossy fiber sprouting and amplifies recurrent excitation within the dentate gyrus-to-CA3 network. A functional consequence of this reorganized circuitry is impairment of dentate gating, i.e., the ability of the dentate gyrus to enforce sparse granule cell firing and restrict downstream CA3 recruitment, thereby facilitating seizure propagation and impairing pattern separation-dependent cognition (Sutula and Dudek, 2007; Le Duigou et al., 2014).

Translating these mechanistic hypotheses to the clinic suggests that the pathophysiological mechanical properties of the epileptic hippocampus can be viewed as a modifiable disease parameter, rather than merely a passive consequence of seizures. Emerging therapeutic strategies for TLE could therefore focus on modulating ECM mechanics using injectable or implantable biomaterials with defined stiffness, degradability, and ligand presentation, designed to recalibrate the physical microenvironment of hippocampal tissue. For example, controlled delivery of ECM modifying agents such as chondroitinase ABC can disrupt maladaptive CSPG networks and alter the mechanical constraints of the sclerotic hippocampal niche (Zhao and Fawcett, 2013; Singh et al., 2021; Patel et al., 2024), potentially biasing structural plasticity toward more stable integration of newborn neurons. However, experimental manipulation of ECM integrity in rodent models has yielded divergent effects on excitability and seizure susceptibility (Rankin-Gee et al., 2015), underscoring the need for spatially precise and temporally controlled interventions. Future approaches may therefore require combinatorial strategies that integrate targeted biomechanical modulation with pharmacological regulation of mechanotransduction pathways, including mechanosensitive ion channels, to reshape how neurons and progenitor cells interpret pathological mechanical cues. Re-framing TLE as a disorder of both electrical and mechanical dysregulation thus expands the therapeutic landscape beyond seizure suppression alone and highlights new avenues for mitigating progressive circuit reorganization and cognitive decline (Bekbolatova et al., 2024).

### 6.4 The inhomogeneous mechanical properties of Alzheimer’s disease brain tissue

The impact of brain tissue stiffness on the physiological properties of stem cells, neurons and glia is an area of active research in the mechanobiology field at present. Moreover, the methods used to measure cellular, ECM, and tissue viscoelastic properties are key to data interpretation because the resolution and length-scale of measurements are important factors when extrapolating potential biological meaning (Pogoda and Janmey, 2018; Faber et al., 2022; Su et al., 2023; Luciano et al., 2024). For a comprehensive review on the various methods that can be used to measure the mechanical properties of distinct brain regions in living humans versus techniques that are more suited to the characterization of *ex vivo* brain slices, we refer the reader to a recent article by Hou et al. (2025). Atomic force microscopy (AFM) is considered the gold standard contact-based method for measuring mechanical properties of tissue at the (sub)micron length scale (Moeendarbary et al., 2013; Marrese et al., 2017). Magnetic resonance elastography (MRE), on the other hand, is the gold standard non-contact method for measuring tissue mechanics at lower resolutions *in vivo* and can be easily performed in living humans (Venkatesh and Ehman, 2014; Yin et al., 2018; Daugherty et al., 2020). Brain tissue viscoelasticity measurements obtained using MRE, however, are often an order of magnitude higher than corresponding AFM measurements. A recent study by Bertalan et al. (2023) suggests that this large discrepancy between measurement techniques is due to viscoelastic dispersion, i.e. the increase in tissue stiffness with higher stimulation frequencies. MRE in the human brain is usually performed using shear wave speeds (SWS) of 30–60 Hz, whereas MRE in the mouse brain requires SWS frequencies of approx. 1 kHz to account for the smaller field of view. In contrast, when performed on *ex vivo* brain slices, AFM uses quasi-static deformations and delivers much lower stiffness measurements than MRE. Both methods generally agree that the mammalian brain gradually stiffens from embryonic phases to neonatal periods to adolescence and peaks in young to mid-adulthood (Ryu et al., 2021). This may be partly driven by increased deposition of ECM proteins and cross-linking of matrix components (Karlinski Zur et al., 2025). In the hippocampal dentate gyrus, this mechanical stiffening correlates with a marked decline in neurogenesis.

In contrast, AFM and MRE measurements of brain tissue stiffness in normal aging are less well correlated and may depend on species (human versus mouse), brain region (dentate gyrus versus neocortex), or myelination levels (white versus gray matter areas). AFM measurements in mice, for example, generally show that brain tissue stiffness either levels off or continues to increase in old age in regions such as the neocortex, striatum, CA1 and dentate gyrus (Segel et al., 2019; Hall et al., 2023). Over a lifetime, however, MRE measurements in humans tend to follow an inverted U shape with tissue viscoelasticity declining from around 40 to 90 years of age (Sack et al., 2011; Arani et al., 2015; Takamura et al., 2020; Delgorio et al., 2021, Hiscox et al., 2021). Alzheimer’s disease (AD), on the other hand, is a neurodegenerative disorder characterized by accelerated volume reductions and softening of whole brain regions, both in humans and rodent models (Menal et al., 2018; Hiscox et al., 2020; Hall et al., 2023; Pavuluri et al., 2025; Träuble et al., 2025). Rather counterintuitively, this significant decrease in tissue viscoelasticity may be caused by the gradual accumulation of thousands of very rigid (10^5^–10^9^ Pascal range) microscopic extracellular amyloid-beta 1–42 (Aβ_42_) plaques and intracellular hyperphosphorylated tau tangles (Mattana et al., 2017). Aβ_42_ plaques and tau tangles disrupt neurotransmission, axonal and synaptic architecture and alter the mechanical properties of neurons and glia (Crimins et al., 2013; Zempel et al., 2013; Spires-Jones and Hyman, 2014; Hu et al., 2023). We have shown that astrocytes may be able to detect the local stiffening of their microenvironment, caused by Aβ_42_ plaque accumulation, by upregulating Piezo1 channels (Velasco-Estevez et al., 2018). Although these peptide and protein aggregates likely increase local tissue stiffness at micron length-scales, they also promote chronic neuroinflammation, microglial reactivity/phagocytosis, and neurodegeneration which leads to the opposite mechanical effect at macro length-scales, i.e., a decrease in global tissue stiffness at millimeter length-scales (Hall et al., 2023; Hu et al., 2023). Recent studies have shown that AHN is significantly reduced in AD patients, with immature neurons exhibiting impaired maturation and integration (Li B. et al., 2008; Tobin et al., 2019; Salta et al., 2023). This could be partly due to an increase in Aβ_42_ induced microglial phagocytosis of SGZ neural progenitor cells (D’Andrea et al., 2004; Cunningham et al., 2013; Kreisel et al., 2019; Diaz-Aparicio et al., 2020; Cutler et al., 2025, *preprint*). Mechanistically, a decrease in DG neurogenesis could be caused by a dysregulation to mechanotransduction and calcium signaling leading to disrupted YAP/TAZ activity in neurogenic niche-associated microglial cells. Interestingly, increased substrate stiffness modulates the Hippo pathway in microglia, causing translocation of YAP to the nucleus and upregulation of the anti-inflammatory cytokine IL-10 and increased proliferation of glioma cells (Fang et al., 2025). However, chronic IL-10 production by astrocytes has been shown to impair hippocampal neurogenesis (Sanchez-Molina et al., 2022). Aβ_42_ instead reduces YAP expression in microglia and leads to a proinflammatory phenotype (Qing et al., 2020). Thus, in the aged Alzheimer’s disease brain there is a plethora of biochemical and mechanical disruptions to hippocampal tissue, and specifically to neural cells in the SGZ of the dentate gyrus, that impact NSC proliferation, differentiation and neurogenic capacity.

**FIGURE 4:**
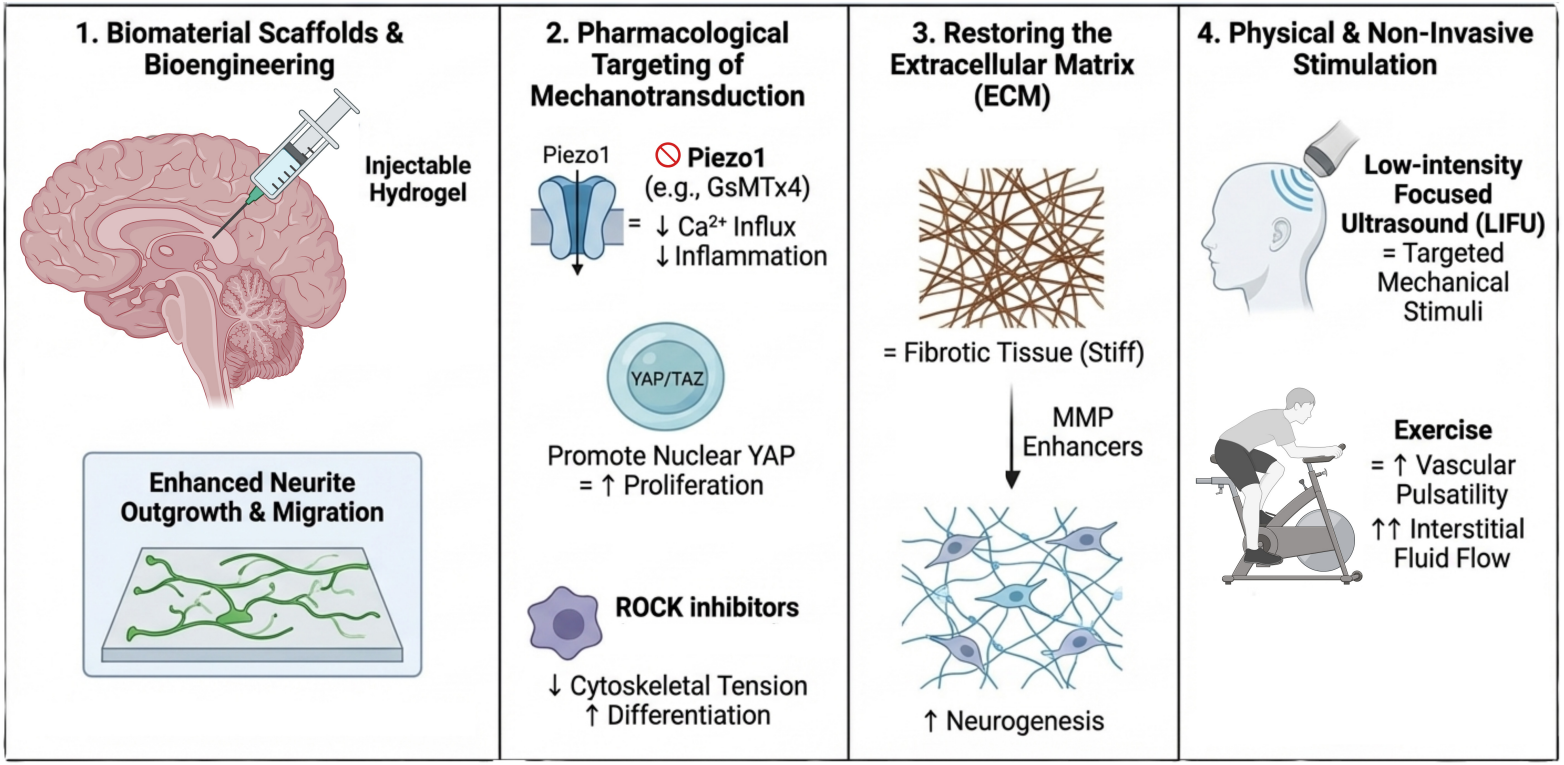
Schematic diagram summarizing several advanced therapeutics that could potentially target the mechanical properties of the neurogenic niche and act to direct neural stem cell fate and promote hippocampal neurogenesis.

Mechanobiology-inspired therapies for AD are currently being developed and trialed (Tobey et al., 2020). In addition to drug-infused or siRNA-releasing hydrogels that manipulate molecular pathways in NSCs (Nguyen et al., 2019; Mukherjee et al., 2020; Hu et al., 2024), non-invasive therapies that deliver targeted mechanical stimulation to the SGZ of the DG may influence neurogenesis through mechanobiological mechanisms. These include techniques such as low-intensity focused ultrasound, transcranial magnetic stimulation, and patterned vibration (Guo et al., 2017; Poon et al., 2017; Cariati et al., 2021; Arulpragasam et al., 2022; Darmani et al., 2022; Seo et al., 2023; Issa et al., 2026), although these therapeutic approaches remain in the early stages of investigation. Such interventions may work by mimicking the natural neurogenesis-promoting properties of physical activity and exercise which are known to enhance AHN and cognitive function, in part, by increasing vascular pulsatility, promoting ECM turnover, and altering interstitial fluid dynamics (Choi et al., 2018; Vecchio et al., 2018; Vonderwalde and Kovacs-Litman, 2018; Yu et al., 2021; Zhao, 2024). As discussed, these biomechanical processes can activate mechanosensitive pathways in NSCs and neighboring cells to stimulate regenerative processes and enhance the release of biochemical factors that regulate neurogenesis. Whether through biomaterials, pharmacological agents, or physical interventions, it may be possible in the near future to restore neurogenesis in the aging, injured or neurodegenerating brain by manipulating the mechanical properties of the dentate gyrus or by targeting the intracellular machinery that interprets these neurogenic signals (Figure 4).

Taken together, many of these recent findings underscore the critical role of mechanobiology in both the maintenance and disruption of hippocampal neurogenesis. Whether through gradual stiffening in adulthood, acute mechanical trauma in TBI or SCI, chronic neural network remodeling in epilepsy, or gradual accumulation of tissue-softening peptide/protein aggregates in AD; alterations in the mechanical microenvironment can profoundly influence NSC behavior and regenerative capacity. As such, targeting the mechanical properties of the neurogenic niche, either through biomaterials, pharmacological modulation of mechanotransduction pathways, ECM-remodeling therapeutics, or targeted ultrasound-based interventions, represents a promising avenue for therapeutic innovation in a range of neurological diseases.

## 7 Comparative mechanobiology: remaining challenges and future directions

Despite recent rapid progress in our understanding of the mechanobiology of adult hippocampal neurogenesis, the field remains in its infancy with numerous conceptual and technical challenges yet to be overcome. At the same time, this nascent field offers a wealth of opportunities for discovery through interdisciplinary collaboration, particularly as new tools emerge to probe the mechanical microenvironment of the brain with increasing precision. A comparative and integrative approach, spanning species, scales, and systems, will be essential to fully elucidate how mechanical forces shape neurogenesis and to translate these insights into therapeutic strategies. While a large proportion of mechanobiological research has focused on rodents, the mechanical properties of the human hippocampus in aging and disease are less well characterized. Comparative studies of the neurogenic niches across species and brain regions, such as songbirds or zebrafish, may illuminate distinct mechanical cytoarchitectures that support lifelong neurogenesis (Polomova et al., 2019; Diotel et al., 2020; Valamparamban and Spéder, 2023). Understanding these differences could reveal conserved versus species-specific mechanobiological mechanisms and inform the design of more effective regenerative therapies.

One of the most pressing challenges lies in the *in vivo* characterization of mechanical properties within the human neurogenic niche. While techniques such as atomic force microscopy (AFM) and traction force microscopy have provided valuable insights *in vitro* and in *ex vivo* tissue slices (Moeendarbary et al., 2017; Colin-York et al., 2019; Hall et al., 2023), measuring stiffness, viscoelasticity, and dynamic mechanical forces in the intact, living brain remains technically demanding (Koser et al., 2016). Advances in magnetic resonance elastography (MRE) have begun to address this gap, offering non-invasive, whole-brain maps of tissue stiffness with submillimeter resolution (Murphy et al., 2019; Hiscox et al., 2021). Ryu et al. (2021) used a similar technique known as ultrasound-based shear-wave elasticity imaging to demonstrate how age-related stiffening of the hippocampus correlates with a decline in neurogenesis. However, further refinement is needed to resolve the microstructural inhomogeneity of the SGZ and to capture rapid, activity-dependent changes in mechanical properties.

Equally important is the need for multi-scale integration, linking molecular-level mechanosensing events to cellular behaviors and tissue-level mechanics (Mak et al., 2015). Mechanotransduction is inherently hierarchical, i.e., primary cilia, integrin molecules, and Piezo channels detect nanoscale deformations which are then transduced through cytoskeletal networks and nuclear mechanosensors to regulate gene expression (Cao et al., 2024; Long et al., 2025). Bridging these scales requires not only experimental innovation but also computational modeling capable of simulating force transmission across cellular compartments and predicting emergent behaviors. Finite element models, agent-based simulations, and machine learning approaches are increasingly being applied to this problem, but their integration with empirical data remains a work in progress (Alber et al., 2019; Mohammad et al., 2025). A further complexity arises from the synergistic and often redundant nature of mechanosensing pathways. For instance, integrins, Piezo1, TRPV4, and primary cilia all respond to overlapping mechanical stimuli, yet their downstream signaling cascades can diverge or converge depending on context (Lee et al., 2015; Servin-Vences et al., 2017; Swain and Liddle, 2021; Cheng B. et al., 2023; Cheng D. et al., 2023). Understanding how these pathways interact, whether through direct cross-talk, feedback loops, or shared transcriptional targets, is essential for deciphering the language of cellular mechanotransduction. For example, how do Piezo1-mediated calcium influx and integrin-FAK signaling coordinate to regulate YAP/TAZ activity in NSCs? Moreover, how do mechanical and biochemical signals (e.g., agonist-mediated G protein-coupled receptor activity) integrate to produce coherent cell fate decisions? These questions remain largely unanswered.

Finally, the mechanobiology field will need to develop ways to integrate complex multicellular experimental data from all cell types within the SGZ of the dentate gyrus, such as astrocytes, microglia, endothelial cells, and pericytes which are all mechanosensitive and contribute to the mechanical and biochemical regulation of AHN. For instance, activated and highly motile microglia can secrete ECM-modifying enzymes and exert contractile forces that may alter local parenchymal stiffness (Crapser et al., 2021; Tewari et al., 2022), potentially impairing neurogenesis during neuroinflammation. Similarly, astrocytic endfeet regulate perivascular mechanics and may influence NSC behavior through both direct contact and paracrine signaling (Petzold and Murthy, 2011; Filosa et al., 2016; Cohen-Salmon et al., 2025). Understanding the intercellular mechanical dialogue within the niche will be essential for constructing a holistic model of hippocampal mechanobiology. Thus, the future of hippocampal mechanobiology lies in embracing complexity across scales, cell types, and disease contexts. With the continued development of advanced imaging, biomaterials, and computational tools, the field is poised to uncover new principles of brain plasticity and regeneration. These insights will not only deepen our understanding of how the brain senses and responds to its physical environment but also pave the way for innovative therapies that harness mechanical signals to restore cognitive function and neural health.
